# Diversity of Intestinal Macrophages in Inflammatory Bowel Diseases

**DOI:** 10.3389/fimmu.2015.00613

**Published:** 2015-12-07

**Authors:** Anja A. Kühl, Ulrike Erben, Lea I. Kredel, Britta Siegmund

**Affiliations:** ^1^Division of Gastroenterology, Infectious Diseases and Rheumatology, Medical Department, Charité – Universitätsmedizin Berlin, Berlin, Germany; ^2^Research Center ImmunoSciences, Charité – Universitätsmedizin Berlin, Berlin, Germany

**Keywords:** intestinal macrophages, gut homeostasis, inflammatory bowel diseases, fibrosis, diversity

## Abstract

Macrophages as innate immune cells and fast responders to antigens play a central role in protecting the body from the luminal content at a huge interface. Chronic inflammation in inflammatory bowel diseases massively alters the number and the subset diversity of intestinal macrophages. We here address the diversity within the human intestinal macrophage compartment at the level of similarities and differences between homeostasis and chronic intestinal inflammation as well as between UC and CD, including the potential role of macrophage subsets for intestinal fibrosis. Hallmark of macrophages is their enormous plasticity, i.e., their capacity to integrate signals from their environment thereby changing their phenotype and functions. Tissue-resident macrophages located directly beneath the surface epithelium in gut homeostasis are mostly tolerogenic. The total number of macrophages increases with luminal contents entering the mucosa through a broken intestinal barrier in ulcerative colitis (UC) as well as in Crohn’s disease (CD). Although not fully understood, the resulting mixtures of tissue-resident and tissue-infiltrating macrophages in both entities are diverse with respect to their phenotypes and their distribution. Macrophages in UC mainly act within the intestinal mucosa. In CD, macrophages can also be found in the muscularis and the mesenteric fat tissue compartment. Taken together, the present knowledge on human intestinal macrophages so far provides a good starting point to dig deeper into the similarities and differences of functional subsets and to finally use their phenotypical diversity as markers for complex local milieus in health and disease.

## Introduction

The gastrointestinal tract is the largest immune compartment of the human body. The major function of the intestinal immune cells is to maintain the integrity of the body at the huge interface between external stimuli that include food components and the intestinal microflora. Chronic inflammation in inflammatory bowel diseases (IBD) profoundly alters the composition of all local immune-cell compartments. Macrophages are part of the innate immune system and instrumental in controlling the barrier function in the small and the large intestine. The macrophages integrate signals from their environment, thereby changing their phenotype and function. The present knowledge about intestinal macrophages is predominantly based on mouse studies. Even the finding of the gut as the largest reservoir of tissue-resident macrophages within the body (1) remains to be verified for men. This minireview deliberately restricts to systematic human studies. Only if such data were lacking, we included findings from animal models that might be relevant for the human mucosal surface. Differences in between mice and men will be highlighted. Non-inflamed tissue areas neighboring the inflamed areas in ulcerative colitis (UC) and Crohn’s disease (CD), the main forms of IBD, represent rather homeostatic conditions. Hence, the diversity within the human intestinal macrophage compartment at the level of similarities and differences between homeostasis and chronic intestinal inflammation as well as between UC and CD, including the potential role of macrophage subsets for intestinal fibrosis, will be discussed.

## Intestinal Macrophages in Gut Homeostasis and in IBD

**Figure 1:**
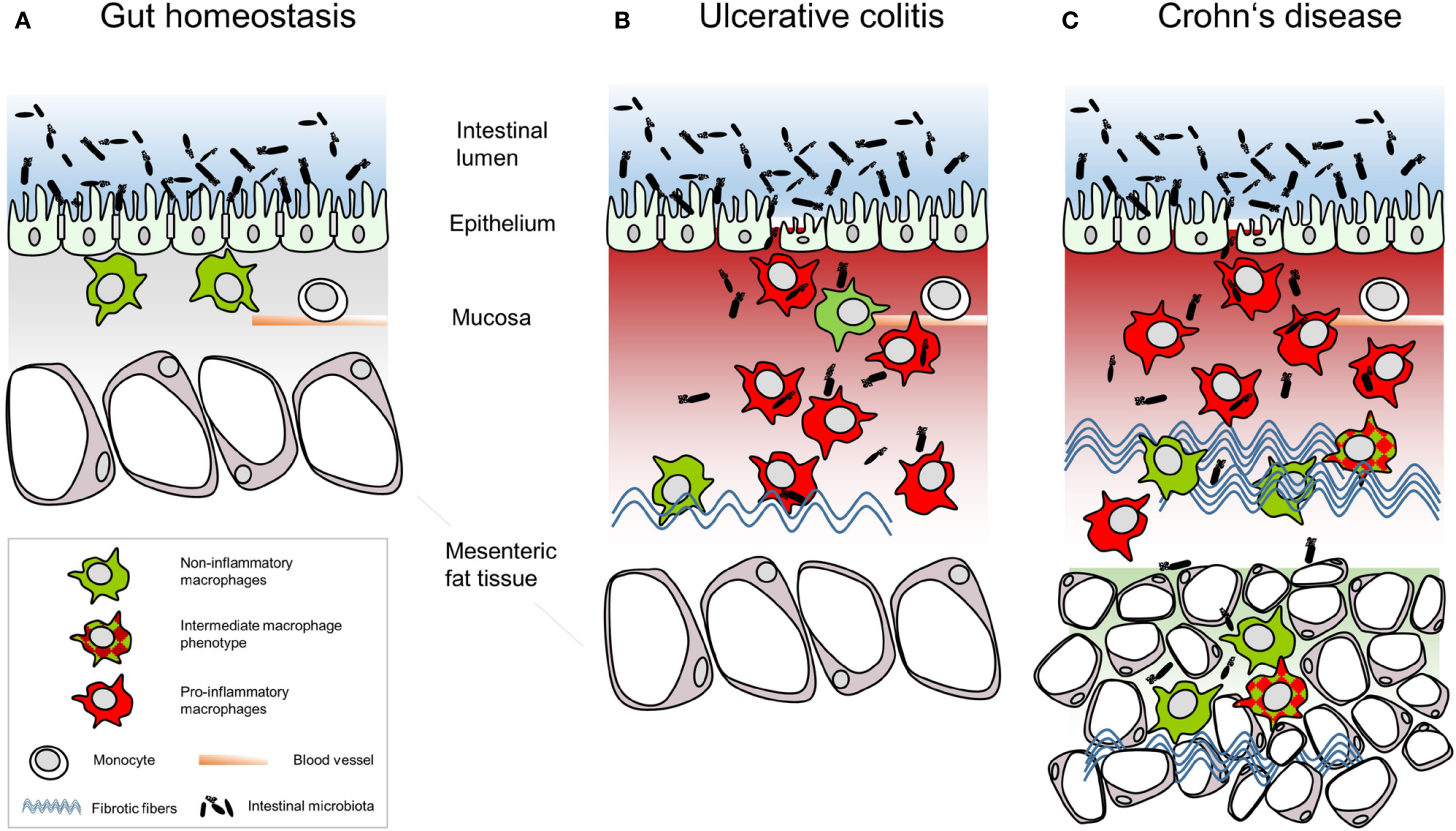
**Schematic summary of the relative intestinal macrophage-subtype distribution in (A) gut homeostasis or (B) ulcerative colitis and (C) Crohn’s disease**.

In terms of a first-line defense, tissue-resident intestinal macrophages contribute to the gut homeostasis by eliminating invading pathogens without inducing an inflammatory response of the lymphocytes within the lamina propria. Positioned directly beneath the surface epithelium, the macrophages in intestinal tissues are the first immune-cell population encountering foreign material, e.g., luminal bacteria or food antigens randomly passing the epithelial barrier (Figure 1A). Whether human macrophages are able to sample luminal antigen by extending their dendrites between the epithelial cells reaching into the gut lumen as shown for mouse macrophages (2, 3) is unknown. On the one hand, intestinal macrophages are tolerant toward foreign matter by down-regulation of recognition receptors (4). On the other hand, intestinal macrophages that recognize food-derived antigens or commensal microbiota present the processed antigens in a tolerizing manner in the absence of co-stimulatory signals (5). Also to fulfill the task of protecting from unwanted immune responses and different from peripheral monocytes, stimulation via pattern recognition receptors (PRR) on resident macrophages results in low cytokine secretion and strong bactericidal activity (6). This increased bacterial clearance is associated with increased metallothionein expression, which is regulated by nuclear factor kappa-light-chain enhancer of activated B cells (NF-κB) and by caspase-1 (7).

Precursors of tissue-resident intestinal macrophages are bone marrow-derived monocytes, which circulate through the blood before recruitment into the intestinal mucosa by interleukin (IL)-8 and transforming growth factor (TGF)β (8). These freshly recruited monocytes exhibit an inflammatory phenotype and exert inflammatory functions. Signals from the intestinal mucosa subsequently polarize them into inflammation anergic macrophages, e.g., by stromal TGFβ-induced inhibition of NF-κB activation (9). Additionally, TGFβ and IL-10 induce down-regulation of triggering receptor expressed on myeloid cells (TREM)-1 on intestinal macrophages, a receptor that potently amplifies inflammatory reactions (10). A minority of tissue-resident intestinal macrophages express CD14 as well as CD11c involved in sensing of bacterial lipopolysaccharides (LPS) and are considered to be differentiation intermediaries (11). Blood monocytes have a life span of 3–4 days, while the life span of intestinal macrophages is unknown. In mice, intestinal macrophages lost upon senescence or apoptosis are constantly replenished by newly recruited blood monocytes and by cell division *in situ* (12). While mouse macrophages replenish in the intestine by recruitment of circulating cells and proliferation (12, 13), human intestinal macrophages fail to do so (8). Again in mice, mucosal tolerance is mediated by intestinal macrophages secreting IL-10, thereby expanding regulatory T cells (Tregs) (14). By contrast, human macrophages isolated from healthy jejunum and stimulated, e.g., with LPS, *Helicobacter pylori* urease, heat-killed *Staphylococcus aureus*, interferon (IFN)γ or phorbol myristate acetate *in vitro* did not produce IL-10 (6).

A hallmark of macrophages is their plasticity as well as the ability to change phenotype and function according to the immediate environment. This has been demonstrated systemically by recent work from Xue and colleagues who defined a core transcriptome network for human and murine macrophages (15).

Hence, it is not surprising that small intestinal macrophages are different from large intestinal macrophages. These two organs have a distinct architecture, exert different functions, and host diverse microbiota. For example, macrophages from healthy jejunum show high expression of human leukocyte antigen (HLA)-DR and very low expression of CD14 and the low-affinity human immunoglobulin (Ig)G receptor CD16 (6), whereas in colonic macrophages low levels of CD14 and CD16 are accompanied by moderately expressed HLA-DR (16). Very early work, e.g., uses the activities of acid phosphatase and nonspecific esterase to distinguish macrophage subtypes (17). Here, tissue-resident intestinal macrophages directly underneath the epithelium differ from macrophages positioned deeper in the lamina propria with no implication that these cells abandon their tolerogenic potential (17).

Following the M1–M2 paradigm, which mirrors the polarization of T helper cells, macrophages are classified as pro-inflammatory M1 macrophages and anti-inflammatory M2 macrophages (18). Adhering to this model, tissue-resident macrophages are ­considered to be M2 macrophages (19, 20). In IBD, macrophages massively infiltrate the intestinal mucosa and present phenotypes and distribution distinct from tissue-resident macrophages in homeostasis. In CD patients, macrophages also infiltrate the muscular layer and the mesenteric fat (17, 21). At first sight, large numbers of CD68^+^ macrophages massively infiltrate the intestinal mucosa in IBD and diffusely spread throughout the thickened mucosa and submucosa but differ with regard to the subset composition and function in UC (Figure 1B) and CD (Figure 1C). Analyses of blood monocytes derived from CD patients reveal a reduction of classical monocytes (CD14^hi^CD16^−^), while intermediate monocytes (CD14^hi^CD16^+^) were increased (22, 23). Extensive migration of classical ­monocytes toward the C-C chemokine ligand (CCL)2 *in vitro* and massively enhanced CD14^hi^ macrophages in the ileal and the colonic mucosa of the CD patients led to the conclusion that peripheral classical monocytes immigrated into the intestinal mucosa (23). These newly recruited macrophages express high levels of CD33, of the high-affinity human IgG receptor CD64 and of the G-protein-coupled fractalkine receptor CX3CR1 but were HLA-DR^dim^ (23). Infiltrating intestinal macrophages are distinct in phenotype and function from their resident counterparts. For example, ­tissue-infiltrating intestinal macrophages strongly express CD14 (24), TREM-1 and the human myeloid IgA Fc receptor CD89 (25) as well as activated NF-κB (26). Additionally, tissue-infiltrating intestinal macrophages secrete pro-inflammatory cytokines such as TNF, IL-6, IL-8, IL-23, IL-1β, and IFNγ as well as the chemokine CCL2 attracting monocytes (25, 27). This pro-inflammatory macrophage phenotype might result from polarization of any monocytic cell entering the pro-inflammatory environment of the inflamed intestinal mucosa. In line with this, the conditioning of newly recruited monocytes toward inflammation anergic M2 macrophages might be disturbed in IBD patients due to defective TGFβ signaling (28). In IBD, a broken epithelial barrier allows luminal content to enter the lamina propria, thereby triggering the inflammatory response of the lamina propria leukocytes. For recognition of microbiota, macrophages up-regulate PRR, including membrane-bound toll-like-receptors (TLR) and C-type-lectin-like receptors (CLR) as well as cytoplasmic nucleotide-binding oligomerization domain-containing protein (NOD)-like receptors (NLR) and retinoic acid-inducible gene-­I-like receptors. Human PRR show less variants than those in mice; 10 TLR and 22 NLR are known in men compared to 13 TLR and 34 NLR in mice. Tissue-infiltrating macrophages in the inflamed colon mucosa predominantly express TLR2, TLR4, and TLR5 responding to bacterial peptidoglycans, LPS, and bacterial flagella (29). CLR bind a variety of carbohydrate ligands but only collectins function in terms of PRR (30). NOD2 recognizing muramyl dipeptide on Gram-positive and -negative bacteria is expressed in monocytes and Paneth cells but not in intestinal macrophages (31). *In vitro* studies showed that NOD2 level declined during differentiation of monocytes into macrophages (31). *CARD15* coding for the caspase-recruitment domain of NOD proteins is highly up-regulated in colonic macrophages of CD patients (32). So far it is not clear whether in chronic inflammation in CD the down-regulation of NOD2 in monocytes infiltrating the colon mucosa is affected or whether resident macrophages up-regulated NOD2 expression. A missense mutation in the coding sequence of *NOD2* was found in 17% of CD patients and in 4% of UC patients (33). As over 200 genes have been linked to IBD (34) and many of them are associated with macrophage functions (35–39), these immune cells present one cell population contributing to the pathogenesis of UC and CD.

## Diversity within Intestinal Macrophage Compartments in Ulcerative Colitis and Crohn’s Disease

Above, we highlighted differences in the macrophage compartments and differentiated between tissue-resident and tissue-infiltrating macrophages in gut homeostasis and IBD. Additionally, the composition and functions of intestinal macrophages also differ in the inflamed gut of UC and CD patients, while overall macrophage numbers are comparable. So the question arises whether distinct macrophage subpopulations and distributions of these subtypes within the inflamed tissue areas might explain the overall different outcome in CD and UC. As for similarities in the local distribution, monocytes and M1 macrophages directly contribute to the defect of the barrier in IBD and large numbers of pro-inflammatory macrophages reside in the inflamed mucosa (40).

Over a decade ago, CD has even been referred to as a macrophage primary immunodeficiency (41). While this statement might simplify the overall interaction of immune cells in the mucosa, several facts add to this hypothesis. Thus, impaired bacterial clearance in CD has been attributed to defective cytokine secretion by macrophages (42). *E. coli* is commonly found within intestinal macrophages in CD (43), a dysfunction not reported for UC. On the contrary, macrophages of UC patients exuberantly and protractedly respond toward bacteria (44). This difference in bacterial clearance is also reflected by the formation of granulomas in CD but not UC (45, 46). Granulomas are formed when the effective eradication of invading pathogens fails.

Tissue-resident intestinal macrophages express the scavenger receptor CD163 that also recognizes Gram-positive and -negative bacteria (47, 48). While CD163 was initially thought to be exclusively expressed on noninflammatory M2 macrophages (49, 50), CD163 is expressed on resident macrophages of all normal tissues except on splenic white pulp macrophages and on germinal center macrophages (51). CD163^+^ macrophages are enriched in the peripheral blood as well as in the colonic mucosa of IBD patients (52–54). As CD163 is cleaved by metalloproteinases (MMPs) and shed from macrophages upon activation, soluble CD163 is an appropriate marker for macrophage activation (55). Compared to healthy controls, sCD163 is increased in UC and CD patients (56). In line with comparable numbers of macrophages in the intestinal mucosa in CD and UC, sCD163 levels are comparable in both entities (56). Upon successful treatment with glucocorticoids or TNFα-antibodies, histomorphologically reflected by reduced macrophages in colon biopsies (57), serum sCD163 levels are reduced (56, 58).

No differences were found regarding the numbers of TREM-1^+^ macrophages triggered to high production of pro-inflammatory cytokines (25) or in the expression of the co-stimulatory molecules CD80 and CD86 (5).

Aldehyde dehydrogenase (ALDH) is involved in the release of retinoic acid, which has immunomodulatory properties and is mandatory in the induction of forkhead-box protein 3^+^ Tregs (59, 60). Directly relating to Treg numbers in the colonic mucosa, ALDH^+^ macrophages are reduced in the intestinal mucosa of UC but not of CD patients (61). While Treg numbers are generally increased in intestinal tissues from IBD patients compared to those of healthy controls, the numbers are lower in UC compared to CD (62, 63). Taking into account that the composition of macrophage subpopulations might mirror the local environment, these findings suggest rather pro- than anti-inflammatory macrophage subpopulations involved in UC.

Specific for CD and relying on the presence of numerous M2 macrophages, the hyperplastic mesenteric fat tissue beyond the transmural inflammation could be defined as a second protective barrier from invading luminal contents (21). In the liver, macrophages are the master regulators of fibrosis (64). Large numbers of macrophages are found in fibrotic lesions of CD patients (65). Gene polymorphisms associated with the fibrostenotic phenotype in IBD like the V249I polymorphism of *CX3CR1* and the T300A mutation in the autophagy-related *ATG16L1* link to macrophage functions (66, 67). An indication for the involvement of distinct macrophage subpopulations in IBD is the development of fibrosis that is more pronounced in CD than in UC (68–70). Fibrosis and subsequent fibrotic strictures result from excessive wound-healing processes. Intestinal wound healing involves various steps with macrophages involved in all of these steps. In the early phase, inflammatory macrophages clear the wound from bacteria and cellular debris; in later phases, wound-healing M2 macrophages promote tissue remodeling. Tissue-resident intestinal macrophages express matrix MMP-2 (71) that takes part in the breakdown of extracellular matrix. In fibrotic CD, MMP2 is increased in the mucosa compared to that of healthy persons (72). The tyrosine-protein kinase Hck, a master regulator for human M2 macrophages (73) regulates myeloproliferation in mice (74). Other studies in mice showed that noninflammatory macrophages are involved at many levels in the whole wound-healing process, i.e., in wound closure, in formation of granulation tissue, in angiogenesis, in collagen synthesis, and in the production of growth factors (75). The pleiotropic cytokine IL-13 was also identified as a pro-fibrotic factor in CD (72). In combination with TNFα, IL-13 induces TGFβ production in macrophages (76).

Macrophages carrying the mannose receptor CD206 and considered wound-healing macrophages (77) are increased in the injured mucosa of UC patients (78). The expression of the proto-oncogene protein Wnt1 by CD206^+^ macrophages enhanced the proliferation of stem cells in response to the epithelial injury in UC (78). Relating to the increased risk of cancer development upon long-standing IBD, large numbers of CD206^+^ macrophages are found in colorectal cancer (79).

Taken together, many open questions remain with regard to specifics of the involvement of different subpopulations of human macrophages in the pathogenesis and the chronicity of UC and CD. Further dissecting the diversity and the local distribution of functional macrophages in human gut tissues will help to define the clinical relevance of the macrophage subset.

## Author Contributions

AK, UE, LK, and BS summarized the content of the manuscript. AK and UE wrote the manuscript, and BS and LK discussed and edited the manuscript.

## Conflict of Interest Statement

The authors declare that the research was conducted in the absence of any commercial or financial relationships that could be construed as a potential conflict of interest.

## Abbreviations

ALDH, aldehyde dehydrogenase; CD, Crohn’s disease; CCL, C–C chemokine ligand; CLR, C-type-lectin-like receptor; CX3CR1, chemokine (C–X3–C motif) receptor 1; HLA, human leukocyte antigen; IBD, inflammatory bowel disease; Ig, immunoglobulin; MMP, matrix metalloproteinase; NF-κB, nuclear factor kappa-light-chain enhancer of activated B cells; NOD, nucleotide-binding oligomerization domain; NLR, NOD-like receptor; PRR, pattern recognition receptors; TLR, toll-like-receptor; TREM, triggering receptor expressed on myeloid cells; UC, ulcerative colitis.
